# A Review on the Current Knowledge on ZIKV Infection and the Interest of Organoids and Nanotechnology on Development of Effective Therapies against Zika Infection

**DOI:** 10.3390/ijms22010035

**Published:** 2020-12-22

**Authors:** Samanta Gasco, María Ángeles Muñoz-Fernández

**Affiliations:** 1 Instituto de Investigación Sanitaria Gregorio Marañón (IiSGM), 28001 Madrid, Spain; samantagasco@gmail.com; 2 Laboratorio InmunoBiología Molecular (HGUGM), 28001 Madrid, Spain; 3 Spanish HIV-HGM BioBank, 28001 Madrid, Spain; 4 Networking Research Center on Bioengineering, Biomaterials and Nanomedicine (CIBER-BBN), 28001 Madrid, Spain

**Keywords:** Zika, organoids, therapy, nanotechnology

## Abstract

Zika virus (ZIKV) acquired a special relevance due to the pandemic that occurred in the Americas in 2015, when an important number of fetal microcephaly cases occurred. Since then, numerous studies have tried to elucidate the pathogenic mechanisms and the potential therapeutic approaches to combat the virus. Cellular and animal models have proved to be a basic resource for this research, with the more recent addition of organoids as a more realistic and physiological 3D culture for the study of ZIKV. Nanotechnology can also offer a promising therapeutic tool, as the nanoparticles developed by this field can penetrate cells and deliver a wide array of drugs in a very specific and controlled way inside the cells. These two state-of-the-art scientific tools clearly provide a very relevant resource for the study of ZIKV, and will help researchers find an effective treatment or vaccine against the virus.

## 1. Introduction

### 1.1. Current Knowledge on Zika Virus and Epidemiology

Zika virus (ZIKV) is a member of the Flaviviridae virus family and the Flavivirus genus, together with other well-known viruses, such as West Nile, dengue (DENV), and yellow fever viruses [1]. It has a single serotype, but two differentiated lineages: The East/West African and the Asian genotypes [2]. It did not present much relevance in relation to human viral infections when it was first isolated from a sentinel rhesus monkey in the Zika forest (Uganda, 1947), as the majority of infections happened in nonhuman primates, and human infections occurred only sporadically [3]. ZIKV was first isolated from mosquitoes in 1948 [3], whereas the first isolation from a human dates back to 1952 [4]. However, the pandemic that occurred in the Americas in 2015 brought ZIKV into the spotlight, mainly due to the important alterations, especially microcephaly, observed in newborns of mothers infected by the virus [1]. It is suspected that this increase in infectivity and severity of clinical symptoms might be due to genetic mutations acquired over time. Concretely, a punctual change of the aminoacid at position 139 of prM, which changed from a serine (S) to an asparagine (N), coincided with the first outbreak on 2013 and might be even related to the increase in fetuses and newborn microcephaly cases in the 2015 outbreak [5,6].

ZIKV is a single-stranded RNA virus that possesses a genome of around 11 kb, which is translated into a big polyprotein encoding 10 viral proteins. Three of these proteins are structural proteins aimed at assembling the viral particles (capsid (C), membrane (M) and envelope (E) proteins), whereas the other seven are nonstructural proteins used by the virus to orchestrate viral replication, avoid host defenses and use the cell machinery to its advantage [7,8]. The viral genome is associated and protected by the icosahedral capsid, which is further surrounded by a glycoprotein envelope composed by over a hundred protein E and M molecules [9]. Protein E is considered to be the most important, as it is the most abundant in the virus surface and has a big relevance in the search for vaccines or neutralizing antibodies against ZIKV infection [8,10,11]. The infectivity ability of ZIKV mostly relies upon prM, E, NS1, NS3 and NS5 proteins and, while some of these proteins are very similar across different members of the Flavivirus Genus, prM, E and NS3 in ZIKV are significantly different to those present in other members of the genus, consequently and possibly explaining the differences in the transmission routes, cellular tropism, tissue persistence and other biological traits [10,12].

### 1.2. Pathophysiology

The main transmission route for infection is through a mosquito vector, namely mosquitoes from the Aedes genus, especially *Aedes aegypti*, which feeds almost exclusively on human blood [9,13]. However, and unlike other flaviviruses, ZIKV is able to transmit from human to human without the help of an insect vector, even if this is not the major route of transmission. This feature potentially responds to the fact that ZIKV is more thermostable than other viruses of the Flavivirus genus, such as DENV, potentially due to ZIKV presenting a more compact structure, which makes it possible to find it in diverse fluids of the human body, like semen, saliva, breast milk or urine, even days or weeks after the disappearance of the clinical symptoms. Hence, some cases of ZIKV infection have been reported by sexual contact, blood or platelet transfusion, mother-to child transmission during pregnancy and, even more rarely, due to close contact in a single reported case [9,13,14,15].

**Figure 1:**
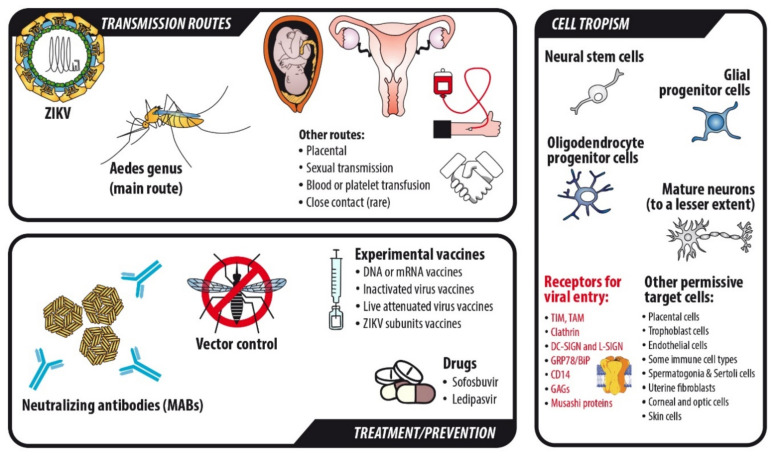
Summary of the transmission routes, cell targets and actual treatments for Zika virus (ZIKV).

The chosen cellular targets of ZIKV are neural-type cells, preferentially neural progenitor cells (NPCs), oligodendrocyte progenitor cells (OPCs) and glial precursors, though there exists some extent of mature neural cell infection too, such as neurons and glial cells [13,16,17,18,19]. However, ZIKV is able to infect other permissive cell types, including placental cells, trophoblast cells, endothelial cells, some immune cell types (such as placental monocytes), spermatogonia and Sertoli cells, uterine fibroblasts, corneal and optic cells and skin cells, among others [7] (Figure 1). The mechanisms for viral entry are not completely known or understood but phosphatidylserine receptors (TIM and TAM protein family members) [20,21,22], clathrin [22], DC-SIGN and L-SIGN [23], GRP78/BiP [24], CD14 [24], glycosaminoglycans (GAGs) [25] and Musashi proteins [26] seem to play a relevant, though not exclusive, role in it.

The life cycle of ZIKV is very similar to that of other viruses from the Flavivirus Genus. The E protein is in charge of initiating the fusion of the mature virions to the host cell membrane, followed by endocytosis into the cell cytoplasm. The viral RNA genome then gets released into the cytoplasm and is translated into the single long polyprotein on the endoplasmic reticulum (ER) membrane. Subsequently, the viral polyprotein gets cleaved to the distinctive 10 proteins (three structural and seven non-structural viral proteins). Maturation of the virions occurs in the trans-Golgi network, where they undergo cleavage of the spiky protein prM-E heterodimer complexes by the protease furin, thus giving rise to the separate M and E proteins, and decreasing the virion particle diameter from ~600 Å to ~500 Å. Maturation is followed by release of the mature virions from the host cell to the extracellular milieu by exocytosis, which is when the pr peptide gets cleaved and released due to the pH returning to a more neutral value [8,9,27]. Interestingly, the huge reorganization suffered by the viral particle during its life cycle was found to be modulated by temperature in the case of DENV, and probably the same could be expected for ZIKV [28].

A very relevant difference of ZIKV with respect to other members of the Flaviviridae family relies on the fact that ZIKV is able to target and provoke neurological symptoms, mostly in fetuses and infants as they have a big population of neural progenitors which is chosen as a target by the virus [29]. Most ZIKV infections are asymptomatic (up to 80% of the total infections). The symptoms encountered, when a symptomatic infection occurs, are usually mild, such as low-grade fever, rash, arthralgia, myalgia, and conjunctivitis. The incubation period usually lasts 4–13 days and the symptoms do not usually last longer than a week. However, in some cases and individuals, severe complications may arise, such as SIKV-associated Guillain-Barré syndrome in adults or congenital Zika syndrome in fetuses and infants born to an infected pregnant women [14].

### 1.3. Current State on the Development of Treatments, Vaccines and Prevention of Zika Infection

There is currently no vaccine or approved small-molecule based drug to prevent or efficiently treat ZIKV infection [30]. Several vaccine candidates have been tested to date in animal models, generating different degrees of immunization against ZIKV infection. They are either based on nucleic acids (DNA or mRNA vaccines), the inactivated virus, the live attenuated virus or subunits of ZIKV [31]. Many of them are currently in phase I clinical trials, with only one of them, a DNA-based vaccine (VRC5283), being in the phase II of a clinical trial at present [31,32]. Research studies have proved that CD8+ cells are the basis of this adaptive immune response against the infection [33], though CD4 T cells, specially the Th1 phenotype, together with IFNγ, could play an indirect role in the establishment of an effective response against ZIKV [34]. Though many of the studied vaccines have shown promising results in vitro and in animal models, including non-human primates [30,31], there are some limitations to these results, as the effectiveness cannot be directly extrapolated to humans, given that the human immune system has its own particularities. In addition, the degree of protection could be different in the different stages of human life (from fetuses to the elderly). Therefore, the vaccine should be carefully designed so that the most vulnerable human group attacked by ZIKV, i.e., pregnant women, are safe when they get the vaccine administered. In addition, it is difficult to test the candidate vaccines given that cases are very rare nowadays. Another added issue is that it is very important to test if antibody-dependent enhancement (ADE) of ZIKV infection would be a reality in people with previous contact with other flaviviruses, given that they may already have antibodies against the virus genus [30] (Figure 1). In addition to this, it should be highlighted that pre-existing immunity against ZIKV could also cause ADE upon DENV infection, and this fact was demonstrated by several studies, in both animal [35,36] and human models [37].

Given the actual limitations on vaccine design against ZIKV infection, passive vaccination by using monoclonal neutralizing antibodies (MAbs) is consequently one of the most thoroughly studied molecules to try and treat the infection. However, most of MAbs tested to date have shown a weak response against ZIKV, which, again, could create the risk of developing ADE related to ZIKV infection [38,39], as well as ADE upon DENV infection if the individual is pre-immunized to ZIKV [40,41,42]. Some MAbs targeting viral protein E have shown promising results, as in the case of C8 and A11, which target quaternary-dependent epitopes in a conserved region of both DENV and ZIKV [43]. One of the main culprits to finding reliable and effective neutralizing antibodies against ZIKV relies on an event known as virus “breathing”, which is a special feature of flaviviruses consisting on the rearrangement of E proteins in the virion surface. This “breathing” can either hide or expose the binding targets of MAbs against ZIKV, making it more difficult to develop an effective vaccine [44,45].

Finally, a huge variety of drug compounds have been tested both in silico and in vitro to date [46], with different potential effectiveness for each of them, but none of them has been implemented in the treatment of ZIKV, so the search for a helpful compound is still ongoing.

A drug that has been recently arising interest in the fight against ZIKV infection is sofosbuvir. Sofosbuvir is a molecule approved for the treatment of hepatitis C virus (HCV) infection that targets and inhibits RNA-dependent RNA polymerase (RdRp). It is usually combined with ledipasvir and is considered a class B drug, thus not being safe for pregnant women. It has shown a promising antiviral effect against ZIKV in vitro and in vivo, as most flaviviruses share a very similar RdRp domain, but it has not been tested on humans in clinical trials. However, given that they this drug is not suitable for pregnant women, its potential usefulness would be restricted to preventing chronic persistence of ZIKV in men and non-pregnant women or used as a prophylactic on high-risk people expending short periods of time in endemic areas of ZIKV infection [47,48] (Figure 1). As there has been little progress made in the prophylaxis and treatment of ZIKV infection, most of the strategies used to prevent new epidemics are based on the control of the mosquito vector [49].

## 2. Cellular and Animal Models used to Date to Study ZIKV Infection, Treatment and Prevention

**Figure 2:**
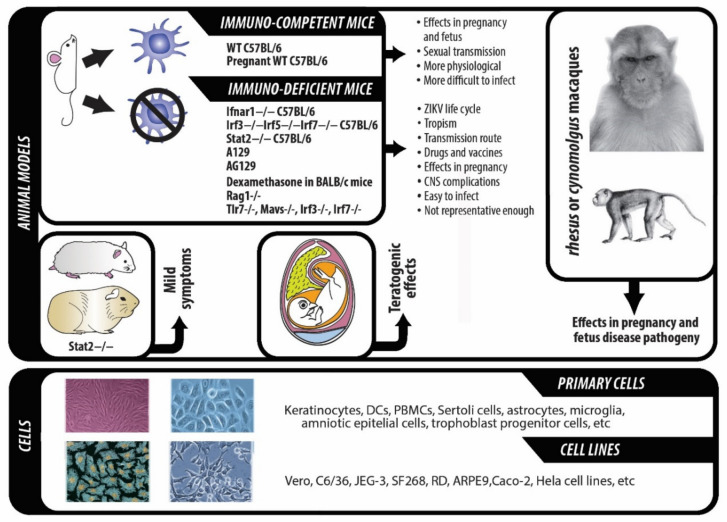
Cellular and animal models used to date for the study of ZIKV features, pathogenic mechanism, transmission routes and tropism.

Since the big outbreak in 2015–2016, many cellular and animal models have been used to study and develop drugs and vaccines against ZIKV infection, to understand its pathogenic mechanisms, transmission routes, life cycle and tropism, among biological aspects of the virus (Figure 2). In general terms, infection with ZIKV has been achieved in most of the animal models tested. However, the severity of the clinical disease induced in the different models tested depends on a wide array of factors, such as age, viral strain, viral load used, transmission route, and others.

### 2.1. Cellular Models

A wide array of human primary cells and cells lines have been used to better understand the pathogenic mechanisms and tropism of ZIKV. Primary cell infection has been proved for neonatal keratinocytes and dendritic cells (DCs), adult DCs, Sertoli cells, amniotic epithelial cells (AmepCs), trophoblast progenitor cells, placental fibroblasts, cytotrophoblasts (CTBs) and human endothelial stromal cells, astrocytes and microglial cells [50].

With regard to cell lines, diverse human cell lines, as well as nonhuman primate cell lines and small animals cells lines have been tested to date with different applications and aims, from studying the replication cycle of ZIKV to testing new potential drugs [51,52]. With respect to human cell lines, some allowed ZIKV replication and suffered from cytopathic effects (CPE) (such as Caco-2, JEG-3 or SF268 cells, among others), whereas others did not show CPE even when the virus replicated effectively within them (such as LNCaP, Hela or Hek cells, among others) [51]. Mosquito-derived cell lines have also been used to study basic questions on the life cycle of the virus, using both *Aedes aegypti-* and *albopictus*-derived cell lines [53,54].

### 2.2. Animal Models

Animal models have been developed to study the effects on the different risk populations. Thus, some strains focused on ZIKV infection on adults and neonates, others aimed at studying the virus effect during pregnancy, while some others targeted the study of sexual transmission, which is quite unique within the Flavivirus Genus [55,56].

Mice models are one of the most used ones for the study of ZIKV life cycle, tropism, transmission route and effectiveness of drugs as well as vaccines. Most mice models generated are based on the concept that genetically altering the type I IFN pathway genes makes mice very susceptible to the Flaviviridae Family (as well as other Arboviruses) infections. This pathway is crucial to defend mammals against RNA viruses by upregulating many IFN-stimulated genes (ISGs), to try and stop viral replication and spread [57,58,59]. Thus, several mutated mice models were generated to try and make them mimic human ZIKV infection, such as *Ifnar1^−/−^* C57BL/6 (IFN-α/β receptor-deficient mice) [60,61], *Irf3^−/−^*;*Irf5^−/−^*;*Irf7^−/−^* C57BL/6 (mice deficient in three regulatory factors of IFN genes) [60], *Stat2^−/−^* C57BL/6 (deficient in a transcription factor that regulates induction of IFN-stimulated genes) [62], A129 (lacking receptors for IFN-α/β as well as receptors for IFN-γ (type II interferon)) [63,64] and AG129 (lacking the IFN-α/β (type I interferon) receptor) [61,64] mice models. *Ifnar1^−/−^* C57BL/6 and *Irf3^−/−^*;*Irf5^−/−^*;*Irf7^−/−^* C57BL/6 mice models showed symptoms of ZIKV infection after inoculating a similar amount of ZIKV injected by the mosquito. Symptoms observed included hunching, limb weakness, ruffled fur and paralysis, with infection even getting lethality depending on the dose and location of inoculation [60]. Interestingly, pregnant *Ifnar1^−/−^* mice that were crossed with C57BL/6 mice also showed fetal development problems leading to fetal demise due to infection of the placenta and the fetus brain [61], so this model is also useful for the study of ZIKV vertical transmission. The *Ifnar1^−/−^* mice were also used by other authors to study which cells in the CNS were more susceptible to ZIKV invasion. Astrocytes were demonstrated to be the most susceptible cell type to infection, while the cerebral cortex neurons were the most resistant ones [65]. The other three mice models also showed different levels of pathology after virus inoculation, that could sometimes lead to severe symptoms [62,63,64]. The A129 mouse model was one of the first ones used to study ZIKV infection [63], while AG129 showed a more severe symptomatology to the one observed in A129, due to the lack of both type I and type II IFN receptors [64,66]. AG129 has been more habitually used for studies on virus persistence in the tissues, as well as for CNS complications that might appear in humans, such as Guillain-Barre syndrome [64]. In addition, a study using the A129 mouse model revealed relevant differences in symptom severity between ZIKV lineages (African or Asian), with the African genotype usually causing a bigger inflammatory response than the Asian genotype, and leading to a different immune profile after infection onset [67]. Interestingly, the outbreak in 2015 in the Americas was predominantly caused by the Asian genotype, which raises a lot of questions as to why this could be, based on the mice results.

Immunosuppression of the immune system by dexamethasone in BALB/c mice is another method to generate useful mice models for the study of ZIKV impact. Inoculation of ZIKV in this model resulted in the appearance of some symptoms mimicking the human pathology: viremia, mild weight loss and detection of ZIKV in several tissues [68].

Mice models can also aid in the study on how ZIKV is sexually transmitted, as well as how it affects pregnancy and fetal development. Diverse mice models have been used to study the sexual transmission route of ZIKV. Male *Ifnar1^-/-^* C57BL/6 mice infected with ZIKV, using either subcutaneous or intraperitoneal inoculation displayed inflammation and injury in different tissues of the male reproductive tract, and ZIKV was detected in various cell types. In addition, severe damage on the testis was observed as late as two months after ZIKV infection, which would suggest that ZIKV infection could potentially cause male infertility [69,70]. Inoculation of the virus in male *Rag1^-/-^* mice, which possesses small lymph nodes with no mature B or T cells, caused a persistent infection in the mice, and ZIKV was detected in several cell types of the male reproductive tract, including Sertoli cells, spermatogonia or spermatocytes, among others. ZIKV infection in this model also showed a high risk for the mice to become infertile [71]. Female mice models have also been used to study sexual transmission of ZIKV. Vaginal inoculation in female WT C57BL/6 or *Tlr7^-/-^, Mavs^-/-^, Irf3^-/-^, Irf7^-/-^* mice was not lethal but allowed the detection of the virus in vaginal fluids [72], while it was lethal and a big viral load was detected in several tissues of the reproductive tract in female *Ifnar1^-/-^* mice [72]. With regard to the study of the effects of ZIKV infection during pregnancy and fetus development, pregnant WT C57BL/6 mice models treated with anti-IFNAR antibodies and subsequently inoculated with ZIKV subcutaneously showed evident damage to the fetuses, including intrauterine growth restriction and detection of the virus in the fetus brain [61]. In the case of WT C57BL/6 mice without any pre-treatment, but using vaginal ZIKV inoculation, a slight decrease in the fetuses weight was observed in addition to finding ZIKV presence in the fetal brain [72]. Intravenous inoculation on another partially immunocompetent mice model, the SJL mice model, led to the observation of intrauterine growth restriction, cortical neuron decrease, as well as ocular abnormalities in the fetus [73]. Immunocompetent models, while not displaying a very severe clinical disease compared to immunocompromised mice models, can aid in acquiring more knowledge on the mechanism governing fetal demise, transplacental transmission and teratogenic effects of ZIKV infection. Infection with ZIKV virus by a peripheral inoculation route does not cause fetal abnormalities. However, when inoculation was performed intracranially at different time points of pregnancy, as well as some days after birth, mice showed a wide range of abnormalities which closely mimicked those observed in humans [74]. By their part, *Ifnar1^-/-^* mice were inoculated subcutaneously with ZIKV, observing placental infection, as well as fetal resorption, fetal brain injury and neural cell death when using subcutaneous inoculation, and even more severe consequences for the fetus when using the intravaginal inoculation [61].

*Stat2*-deficient hamsters and guinea pigs have also been used to study ZIKV pathogeny, resulting in a mild representation of the disease [75,76]. Chicken embryos can also be used to specifically study the teratogenic effects observed in human fetuses [77].

Nonhuman primates have also been used as evident animal models for the study of the virus life cycle and pathogenesis, as ZIKV also has them as hosts and reservoirs. Subcutaneous inoculation in either rhesus or cynomolgus macaques provoked mild clinical disease with viraemia and persistence of the virus in several tissues of the body, including the CNS, the lymph nodes or the female or male genital tract [78,79,80]. Intravenous inoculation lead to no clinical symptoms in both macaque species [81], while intrarectal or intravaginal inoculation of ZIKV resulted in viremia with clinical disease in rhesus macaques but viremia with no clinical disease in cynomolgus macaques [82]. Infection of pregnant rhesus macaques showed the appearance of lesions similar to those observed in human fetuses suffering from congenital Zika syndrome (CZS), including fetal loss, altered brain size and microscopic brain lesions. In addition, research showed that the viraemia observed in these pregnant females lasted longer in the early months of pregnancy and compared to non-pregnant females [83].

## 3. Organoids for the Study of ZIKV Infection

Organoids are 3D cell cultures that develop and acquire morphological and functional characteristics like those of the organs they try to resemble, given the appropriate conditions to do so [84]. Organoids can, not only help to better understand tissue physiology and formation, but also offer extensive information on the pathogenic mechanisms of relevant human diseases, on potential diagnostic and prognostic biomarkers as well as on drug screening and therapy efficacy, among other applications [84,85,86]. Organoids present several advantages for drug screening that conventional 2D culture and animal models are lacking, as they mimic more accurately the environmental conditions that would be present in the organism, as well representing the communication and relations between the different cell populations of the tissue. They also resemble to a higher level the architecture of the original tissue or organ, which leads to a better approximation on how potential therapeutic agents would enter the organ and act within it [87]. In addition, the creation of special bioreactors has enabled the generation of bigger and more complex organoids, even more closely resembling the layers of the tissue which is being replicated, a fact that is particularly crucial in the case of brain organoids [88]. 2D and animal models have indeed provided a huge knowledge compilation on many factors affecting ZIKV biology and drug mechanisms, but organoids can help complete and boost that knowledge via their specific advantages in comparison to the more classical methods.

With regard to ZIKV infection, brain organoid models have been used to study the pathogenic mechanisms of the infection [14]. Comparison of the growth rates of mock-, DENV- and ZIKV-infected brain organoids showed that there was a reduction of up to 40% in size of mature organoids in the case of ZIKV infection. In addition, the fatal effects produced by ZIKV on hiPSC-derived NSCs, formation of neurospheres and growth of organoids was encountered only for this virus, but not in the case of the organoids infected with DENV. Therefore, these features seem to be specific of ZIKV, not of the whole genus [73,89]. ZIKV was shown to increase neural cell apoptosis, possibly explaining the observed cell number reduction that also contributes to microcephaly [73,90].

**Figure 3:**
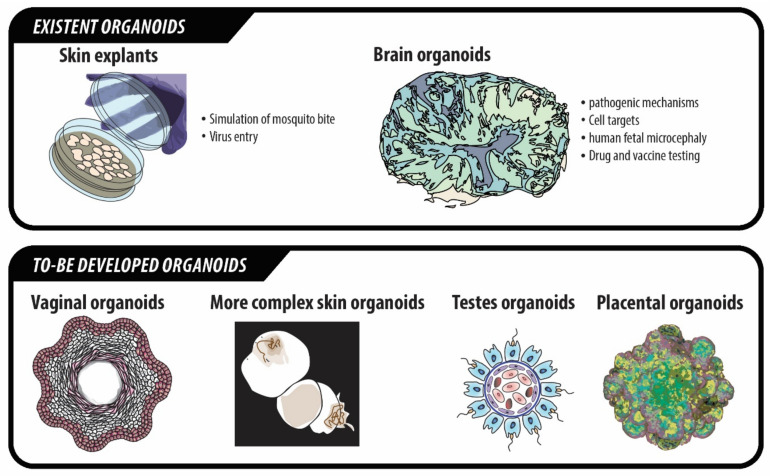
Organoid models used to date and potential for new to-be developed organoids for the study of ZIKV and its treatment and prevention.

Brain organoids generated by using a mini-biorreactor were used to assess the impact of ZIKV infection at different stages of pregnancy. Early-stage organoids simulating the first trimester of human pregnancy were infected and the cells affected by the virus analyzed. It was demonstrated that the virus had a preference to infect NPCs, though it could also target IPCs, immature neurons and even astrocytes. When infecting more complex and aged organoids, more closely resembling the second trimester of pregnancy, specific infection of SOX2+ cells, including HOPX+ outer radial glia cells (oRGs) was observed. All these findings correlated with an increase in cell apoptosis and reduced proliferation, and mimicked some of the features observed in human fetal microcephaly [88]. Using brain organoids, Dang et al. found that the increase in NPCs apoptosis, ultimately leading to microcephaly, was linked to an increase in the expression levels of Toll-Like Receptor 3 (TLR3) in these cells after being infected by ZIKV [91]. TLR3 is a protein which has been demonstrated to be highly expressed during the early stages of brain development. AS NPCs begin to differentiate toward their final fates, the expression levels of TLR3 start to decrease [92,93]. In addition, the use of brain organoids to study ZIKV indicated that the virus can have even more profound effects, even altering DNA methylation in NPCs, astrocytes and mature neurons, which could lead to additional neurological problems for the fetus [94]. Another study also indicated that NS2A protein of ZIKV alters and decreases proliferation of radial glial cells, causes their premature differentiation and also provokes an altered positioning of new neurons [95]. In addition to using brain organoids for the study of ZIKV pathogenic mechanisms, they have also been used to test drug compounds against ZIKV infection, with some of them showing promising results [90,96] (Figure 3).

Though not yet generated, vaginal and placental organoids, as well as testes organoids, would also be very interesting and useful, as ZIKV is the only virus in the flavivirus genus that is able to infect the body through sexual transmission, with transmission from the male to the female being the most usual direction of infection [55,56] (Figure 3).

The use of skin organoids would also aid in the study on how the virus enters the body through the mosquito bite (Figure 3). Skin explants have been used recently to study ZIKV entry (and other flaviviruses entry) through the skin [97,98,99]. However, no literature describes the use of complex skin organoids for this purpose to date.

## 4. Nanotechnology for the Study of Zika Infection

Nanotechnology is the branch of science aiming to generate compounds at the nanoscale range, i.e., 1–100 nM [100]. In the last decades, biomedicine has started to take advantage of this relatively new branch of science, in relation to bioimaging techniques, disease diagnosis and drug development and delivery. Nanotechnology has shown a special relevance in the cancer field, concretely to try and deliver therapeutic compounds to specific targets, instead of introducing them by the usual entrance points and risking the active ingredients to affect other regions or the organisms, which is one of the main risks and side effects of the current chemotherapy and radiotherapy methods against cancer [100].

However, these compounds have also shown a promising potential for infectious diseases, including viral infections like those caused by ZIKV. Given the fact that no vaccine or molecule tested to date has been completely effective or curative against the virus, nanotechnology could offer an alternative approach for the treatment of ZIKV infection, as well as for the prevention and elimination of the mosquito vector [101]. Though the field is still incipient, one of the families of nanotechnology compounds, dendrimers, could offer a potential way of preventing and treating ZIKV infection [102,103]. To date, some studies have been performed in relation to nanoparticles for treatment of ZIKV infections, including one study for the diagnosis of the infection, where a paper microchip with integrated platinum nanoparticles (PtNPs) gave rise to a very interesting and new way of detecting ZIKV in lysates [104]. A RNA nanoparticle vaccine against ZIKV has also been developed and tested in C57BL/6 mice [105].

## 5. Conclusions

In summary, there is a lot that is still unknown about ZIKV, and the occurrence of more prominent epidemics caused by other pathogenic microorganisms has slowed down the advance in ZIKV related research. The cellular and animal models available have shed light on how the virus acts, why it acquired a more aggressive nature in the 2015 epidemics and how to potentially combat it more effectively, but there is still a lot of progress to be made in the development of an effective vaccines, and both organoid cultures and nanotechnology compounds can pave the way to achieve them.
